# All-Organic Semiconductors for Electrochemical Biosensors: An Overview of Recent Progress in Material Design

**DOI:** 10.3389/fbioe.2019.00237

**Published:** 2019-09-25

**Authors:** Jonathan Hopkins, Kristina Fidanovski, Antonio Lauto, Damia Mawad

**Affiliations:** ^1^School of Materials Science and Engineering, University of New South Wales Sydney, Sydney, NSW, Australia; ^2^Centre for Advanced Macromolecular Design, University of New South Wales Sydney, Sydney, NSW, Australia; ^3^School of Science, Western Sydney University, Penrith, NSW, Australia; ^4^Australian Centre for NanoMedicine, ARC Centre of Excellence in Convergent Bio-Nano Science and Technology, University of New South Wales Sydney, Sydney, NSW, Australia

**Keywords:** conjugated polymer, organic semiconductor, electrochemical, biosensor, material design

## Abstract

Organic semiconductors remain of major interest in the field of bioelectrochemistry for their versatility in chemical and electrochemical behavior. These materials have been tailored using organic synthesis for use in cell stimulation, sustainable energy production, and in biosensors. Recent progress in the field of fully organic semiconductor biosensors is outlined in this review, with a particular emphasis on the synthetic tailoring of these semiconductors for their intended application. Biosensors ultimately function on the basis of a physical, optical or electrochemical change which occurs in the active material when it encounters the target analyte. Electrochemical biosensors are becoming increasingly popular among organic semiconductor biosensors, owing to their good detection performances, and simple operation. The analyte either interacts directly with the semiconductor material in a redox process or undergoes a redox process with a moiety such as an enzyme attached to the semiconductor material. The electrochemical signal is then transduced through the semiconductor material. The most recent examples of organic semiconductor biosensors are discussed here with reference to the material design of polymers with semiconducting backbones, specifically conjugated polymers, and polymer semiconducting dyes. We conclude that direct interaction between the analyte and the semiconducting material is generally more sensitive and cost effective, despite being currently limited by the need to identify, and synthesize selective sensing functionalities. It is also worth noting the potential roles of highly-sensitive, organic transistor devices and small molecule semiconductors, such as the photochromic and redox active molecule spiropyran, as polymer pendant groups in future biosensor designs.

## Introduction

Bioelectrochemistry is the study of naturally-occurring, reduction/oxidation (redox) processes in living systems, encompassing electron transfer in biomolecules, enzyme redox behavior at an electrode, and interactions between synthetic, electro-responsive materials, and biological systems (Wu et al., 2017; Cervera et al., 2018; Oliveira-Brett et al., 2019). Recently, these natural electrochemical processes have been harnessed in sensors to detect molecules, including biomolecules relevant to the diagnosis, and management of human disease (Naveen et al., 2017). Biosensor materials change their physical, optical, or electrochemical properties in the presence of analyte molecules, thus “sensing” the analyte. For example, optical biosensors undergo changes in optical properties, including optical band gap and absorption and emission spectra, upon interaction with an analyte (Alvarez et al., 2011; Wang J. et al., 2018). Inorganic piezoelectric biosensors have been used as immunosensors and pesticide chemosensors (Skládal, 2016; Pohanka, 2017), while calorimetric (thermal) biosensors detect temperature changes from analyte reactions, and include purely-enzymatic thermistors (Antonelli et al., 2008; Bhand et al., 2010), and piezoelectric quartz oscillators (Gaddes et al., 2017). Electrochemical biosensors, which detect redox reactions of analyte molecules as electrical signals, are especially promising with their low cost, high sensitivity and selectivity, and simple apparatus (Aydin et al., 2018; Moon et al., 2018; Chai and Kan, 2019); they are therefore the focus of this review.

To this end, organic semiconductors are of major interest since their chemical and electrochemical properties can be tailored using organic synthesis to the targeted application. They are the core component of organic bioelectronic devices for biomolecule sensing (Park et al., 2008; Wang et al., 2019), cell stimulation (Fidanovski and Mawad, 2019; Hopkins et al., 2019), and sustainable energy generation (Chen et al., 2004; Wallace et al., 2005; Li et al., 2017). In electrochemical biosensors, small molecule semiconductors are used individually or as polymer pendant groups; for example, spiropyran derivatives are photochromic and undergo ring-opening isomerization under various stimuli (Miyagishi et al., 2019), while organometallic “redox polymers” bearing pendant ferrocene, and osmium complexes have detected numerous neurotransmitter molecules (Casado et al., 2016). However, conjugated polymers (CPs)—polymers with π-conjugated, semiconducting backbones—exhibit improved sensitivity in biosensing due to their high electrical conductivity and efficient, tailorable charge transport characteristics, permitting rapid signal transduction (Park et al., 2008), and their biocompatibility allows their biological application (Cevik et al., 2019). Importantly, their redox-active backbone and propensity for flexible modification with numerous chemical functionalities allow them to mediate electrochemical reactions. Similarly, commercially-available, organic dyes such as methylene blue are readily electropolymerized, producing polymers with semiconducting backbones which efficiently mediate charge transport and redox reactions, with demonstrated utility in biosensing (Barsan et al., 2015). Given these polymeric materials' advantages, this review examines the material design of all-organic polymers with semiconducting backbones for electrochemical biosensors. We first discuss the operating principles and mechanisms of electrochemical biosensors. We then review the recent syntheses of novel, organic polymers with semiconducting backbones, including functional CPs and polymers of organic dyes, and outline their electrochemical detection of significant biomolecules.

## Organic Semiconducting Polymers for Electrochemical Biosensors

### Operating Principles of Electrochemical Biosensors

**Figure 1:**
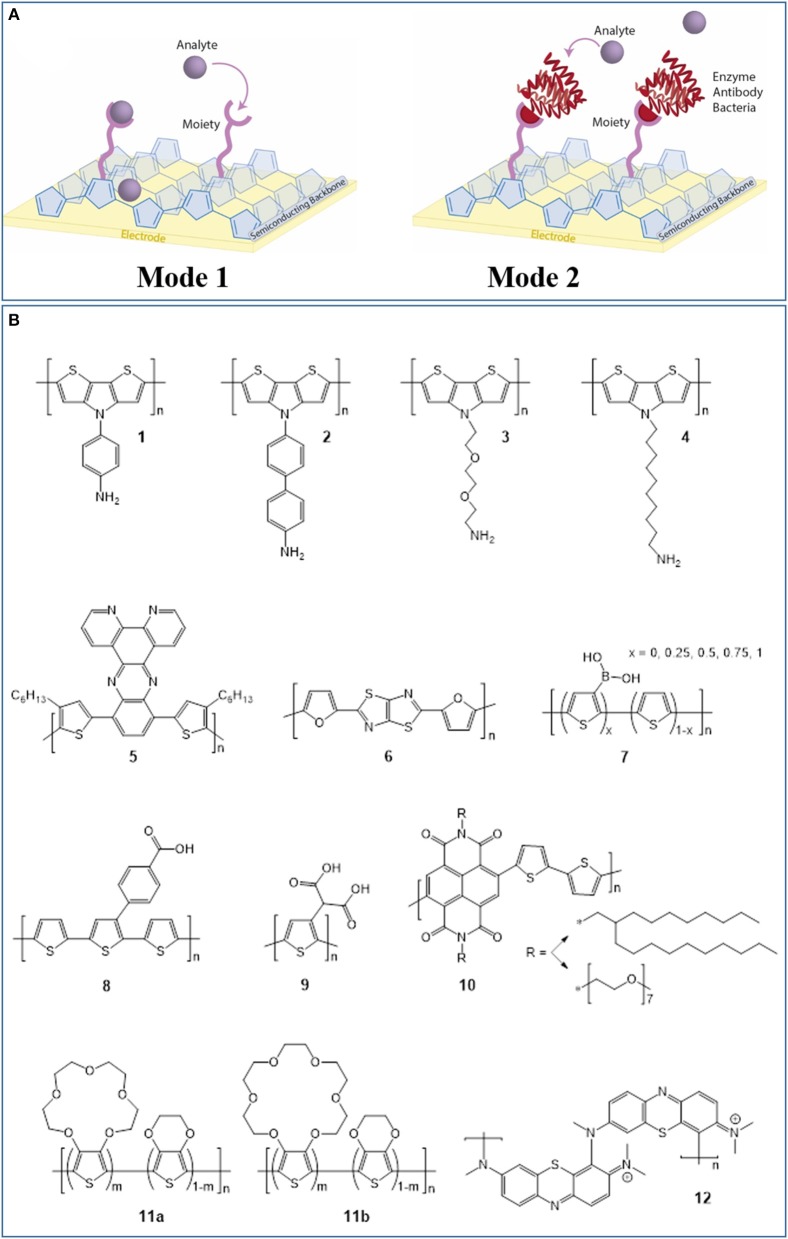
**(A)** The two modes of detection mechanism in organic electrochemical biosensors. Mode-1 detection involves direct interaction between polymer functionalities and the analyte, while mode-2 detection requires additional, biosensing moieties such as enzymes, antibodies, or bacteria to be chemically attached to the polymer. **(B)** Chemical structures of organic semiconductors used in electrochemical biosensors in recent literature.

Electrochemical sensors operate as transducers in an electrochemical cell, facilitating analyte binding or electrochemical reaction at the surface under an applied potential (Park et al., 2008; Moon et al., 2018). This potential is applied using: amperometry, which monitors changes in current at constant potential; potentiometry, which measures potential with no current; impedometry, which measures the steady-state current response to a small alternating potential, typically using electrochemical impedance spectroscopy (EIS); and cyclic voltammetry (CV), which measures the current under a cyclic potential, generating current peaks from redox reactions. Sensor-analyte electron transfer results in electrochemical signals which are transduced through the semiconductor into an underlying electrode, and detected using a potentiostat. Transistor configurations, including organic electrochemical transistors (OECTs) introduced by White et al. (1984), utilize an additional gate voltage to amplify signals and improve biosensor sensitivity, as recently reviewed (Bai et al., 2019; Wang et al., 2019). Electrochemical methods in general offer inexpensive, simple operation, high sensitivity and selectivity, low limits of detection (LOD), and broad linear detection ranges—all important criteria for biosensors (Aydin et al., 2018; Moon et al., 2018; Chai and Kan, 2019). Organic electrochemical biosensors have two modes of detection mechanism (Figure 1A). The organic semiconductor can be chemically modified with functionalities that directly facilitate analyte redox reactions. Alternatively, the organic semiconductor can be functionalized with complex moieties, including enzymes (enzymatic sensors), antibodies (immunosensors), and bacteria (bacterial sensors), which bind to the analyte, and mediate its reduction/oxidation. In this review, we refer to these mechanisms as “mode-1” and “mode-2,” respectively. Both mechanisms require semiconductor materials combining efficient charge carrier transport within the material with novel functionalities for either sensing mode. Consequently, for organic semiconductor-based biosensors, chemical synthesis is important to access novel material properties and functionalities tailored to detecting specific biomolecules. Therefore, we next discuss the recent syntheses of organic polymers with semiconducting backbones for electrochemical biosensors, focusing on conjugated polymers, and polymers of semiconducting organic dyes.

### Material Design for Electrochemical Biosensors

The tailored synthesis of CPs for biosensors has received significant attention in recent literature. In particular, electrochemical glucose biosensors have been prominent since their invention by Clark and Lyons (1962) because glucose is relevant to many diseases, including endocrine disorders, and diabetes (Cevik et al., 2019). For example, the CP poly(dithieno(3,2-b:2′,3′-d)pyrrole) (PDTP) and its derivatives have previously attracted interest in organic field-effect transistors (OFETs), and more recently in electrochemical glucose biosensors, since their planar structures and fused ring systems yield high hole mobilities reaching 0.41 cm^2^ V^−1^ s^−1^ and efficient, enzyme-to-electrode charge transport (Parameswaran et al., 2009; Rasmussen and Evenson, 2013). Azak et al. (2016) synthesized PDTP derivatives bearing *N*-substituted aniline (Figure 1B, semiconductor 1) and biphenylamine (2) functionalities for “mode-2” glucose detection. The dithieno(3,2-b:2′,3′-d)pyrrole (DTP)-based monomers were synthesized using either Cu-catalyzed, Ullmann-type coupling or Pd-catalyzed, Buchwald-Hartwig coupling of 3,3′-dibromo-2,2′-bithiophene with aryl amines. Monomers underwent CV electropolymerization onto a gold substrate, then glucose oxidase (GOx) enzyme molecules, and gold nanoparticles (AuNPs) modified with amine functionalities were immobilized on the polymer surface. AuNPs are commonly incorporated into biosensors to improve sensor sensitivity and selectivity (Naveen et al., 2017; Moon et al., 2018). The GOx catalyzed glucose oxidation in phosphate-buffered solution (PBS, pH = 7.4) during chronoamperometry. Both PDTP derivatives afforded wide linear detection ranges, and polymer 2 gave an especially low LOD of 0.0986 μM (Table 1). The biosensors reliably measured glucose concentrations in spiked human blood samples (<1% relative standard deviation, RSD), emphasizing their potential in diabetes treatment.

**Table 1:** Summary of biosensor configurations, mechanisms, and performances.

**SC**	**Additional species**	**Analyte**	**Detection mechanism**	**Detection method**	**Sensitivity (μA mM^**−1**^ cm^**−2**^)**	**LOD/Linear range (μM)**	**References**
1	GOx, AuNPs	Glucose	Mode-2, enzymatic	CA	NR	50.0/100–2,500	Azak et al., 2016
2	GOx, AuNPs	Glucose	Mode-2, enzymatic	CA	NR	0.0986/50–1,000	Azak et al., 2016
3	GOx	Glucose	Mode-2, enzymatic	CV	NR	0.348/50–900	Azak et al., 2018
4	GOx	Glucose	Mode-2, enzymatic	AM	NR	22/45–50,000	Cevik et al., 2019
4	*G. oxydans*	Glucose	Mode-2, bacterial	AM	NR	81/190–50,000	Cevik et al., 2019
5	GOx	Glucose	Mode-2, enzymatic	AM	105.12	2.88/25–1,000	Buber et al., 2018
6	GOx	Glucose	Mode-2, enzymatic	AM	65.44	12.8/5–700	Soylemez et al., 2019
7	—	Dopamine	Mode-1	EIS	NR	0.3/7.8–125	Dervisevic et al., 2017a
7	—	Sialic acid (AGS cells)	Mode-1	EIS	NR	10/10–10^6^ ^d^	Dervisevic et al., 2017b
8	Acetylcholinesterase/ choline oxidase, AuNPs	Acetylcholine	Mode-2, enzymatic	CA	NR	0.6/0.7–1,500	Akhtar et al., 2017
9	Anti-IL-1β antibodies	Interleukin 1β	Mode-2, immunosensor	EIS	NR	3 × 10^−6^/1 × 10^−5^–0.003^c^	Aydin et al., 2018
10	GOx	Glucose	Mode-2, enzymatic	CA/EIS	NR	10/10–10,000	Savva et al., 2019
10	Lactate oxidase	Lactate	Mode-2, enzymatic	CA	NR	10/10–1,000	Pappa et al., 2018
11a	—	Sodium ions	Mode-1	CA/CV/SS	37^a^	20/10–10^6^	Wustoni et al., 2019
11b	—	Potassium ions	Mode-1	CA/CV/SS	49^a^	100/100–10^6^	Wustoni et al., 2019
12	GDH, NAD^+^	Glucose	Mode-2, enzymatic	AM	NR	4.0/10–1,000	Dilgin et al., 2018
12	—	Creatine	Mode-1	DPV	0.133^b^	0.2/0.5–900^c^	Pandey et al., 2018

SC, Semiconductor; GOx, glucose oxidase; AuNPs, gold nanoparticles; GDH, glucose dehydrogenase; NAD^+^, nicotinamide adenine dinucleotide; SCNs, single-walled carbon nanotubes; CA, chronoamperometry; CV, cyclic voltammetry; AM, amperometry; EIS, electrochemical impedance spectroscopy; DPV, differential pulse voltammetry; SS, steady-state OECT measurements; NR, not reported.

a Units are μA decade^−1^.

b Units are μA ng mL^−1^.

c Units are ng mL^−1^.

d *Units are cells mL^−1^*.

The authors subsequently developed PDTP biosensors without AuNPs (Azak et al., 2018). They synthesized alkoxyamine-functionalized PDTP (**3**) *via* Buchwald-Hartwig coupling and performed CV electropolymerization onto an indium tin oxide (ITO)-glass substrate. The electron-donating alkoxyamine side groups were chosen over alkyl amine groups to reduce the energy requirement for monomer oxidation during electropolymerization. GOx was immobilized by covalently bonding to polymer amine groups, assisted by glutaraldehyde (GA) crosslinking. Glucose was detected in PBS (pH = 7.0) with a low LOD of 0.348 μM, superior to many electrochemical glucose biosensors (Table 1). The bound enzymes exhibited impressive storage stability, retaining 80% enzymatic activity after 20 days—a significant step toward improving biosensor longevity. The same group synthesized *N*-decylamine-substituted DTP monomer by Buchwald-Hartwig coupling, as reported previously (Udum et al., 2014), followed by electrochemical polymerization onto a glassy carbon electrode giving polymer **4** (Cevik et al., 2019). They then fabricated enzymatic and bacterial biosensors for glucose by utilizing the decylamine functionalities to immobilize GOx and *Gluconobacter oxydans* (*G. oxydans*) onto the surface. *G. oxydans* has demonstrated utility in electrochemical biosensors for numerous functionalities (Katrlík et al., 2007). Both biosensors detected glucose *via* amperometry in PBS at optimized pH. While the bacterial biosensor exhibited more reliable detection in the presence of other molecules, the GOx biosensor was superior overall with a lower LOD (22 vs. 81 μM) and broader linear dynamic range (0.045–50.0 vs. 0.19–50.0 mM).

Buber et al. (2018) explored an alternative glucose sensor involving a novel, bithiophene-phenazine-based CP (**5**) without amine functionalities as a GOx-immobilization substrate. The monomer, 10,13-bis(4-hexylthiophen-2-yl)dipyridol[3,2-a:2′,3′-c]phenazine (HTPP), was synthesized by Stille coupling of thiophene and phenazine precursors, phenazine reduction to amines, and condensation with an aromatic dione (Esmer et al., 2011). The polymer was deposited using CV onto a graphite substrate. Structurally, the polymer was designed to improve GOx adhesion *via* hydrophobic interactions with hexyl side chains and intermolecular π-π interactions with the numerous aromatic rings. Through GA crosslinking, GOx was immobilized on the surface to oxidize and detect β-d-glucose in PBS by measuring molecular oxygen consumption using amperometry. After optimization, the sensor exhibited superior sensitivity (105.12 μA mM^−1^ cm^−2^), and LOD (2.88 μM) to comparable published systems, and accurately measured glucose levels in commercial beverages (<10% deviation from product label).

Donor-acceptor CPs, containing electron donor and acceptor repeat units, exhibit efficient electrochromic switching, and applications in OFETs and solar cells (Soylemez et al., 2019), and also possess biosensing capabilities. Soylemez et al. (2019) synthesized a donor-acceptor CP (**6**) containing furan (donor) and thiazole (acceptor) moieties in a “proof-of-concept” glucose biosensor. The monomer, 2,5-di(furan-2-yl)thiazolo[5,4-d]thiazole, was synthesized in a mild, single-step reaction and underwent CV electropolymerization onto a graphite electrode. The polymer exhibited reversible electrochromic behavior with fast redox switching times (0.3 and 0.4 s). After immobilizing GOx with GA crosslinking, glucose was detected in beverages with good sensitivity (65.44 μA mM^−1^ cm^−2^) and reasonable LOD (12.8 μM). Although these values are inferior compared to Buber et al. (2018), this work emphasizes the wide range of CP designs applicable to biosensing. CPs have also been used to detect neurotransmitters including dopamine, a neurotransmitter involved in several neurological conditions including Alzheimer's disease. Dopamine binds strongly and selectively to boronic acids, permitting mode-1 dopamine detection. However, the low physiological concentration of dopamine necessitates high biosensor sensitivity and selectivity (Jiang et al., 2017). As such, Dervisevic et al. (2017a) copolymerized thiophene and 3-thienylboronic acid by CV onto pencil graphite, producing a PT derivative (**7**) with boronic acid groups. In dopamine solution, these groups immobilized dopamine molecules at physiological pH, altering the devices' impedimetric response. This permitted selective dopamine detection in human urine with a wide linear range (7.8–125 μM) and very low LOD (0.3 μM). This CP also selectively detected tumor cells (Dervisevic et al., 2017b): electrodes coated with electropolymerized CP were submerged in a medium containing human Caucasian gastric adenocarcinoma (AGS) cancer cells, which generate abnormally large quantities of sialic acid (1,000× greater). Through EIS, the boronic acid-functionalized CP detected sialic acid with high selectivity and a low cell LOD (10 cells mL^−1^), highlighting its potential in reliable, early cancer diagnosis.

Akhtar et al. (2017) developed an innovative biosensor for the neurotransmitter acetylcholine associated with various neurological and physiological conditions. They used a dual-electrode, microfluidic device to improve enzyme loading, and minimize signal interference. The monomer, 2,2′:5′,2″-terthiophene-3-(p-benzoic acid), was synthesized by boronic acid functionalization of 3′-bromo-2,2′:5′,2″-terthiophene, Suzuki coupling with 4-bromobenzonitrile, and alkaline hydrolysis of nitrile groups to carboxylic acid groups (Kim et al., 2012). This monomer underwent CV electropolymerization, depositing polymer **8** onto an AuNP-coated “reaction electrode” and a porous, Au-coated “detection electrode.” Acetylcholinesterase was immobilized on the reaction electrode and choline oxidase was immobilized on the detection electrode. Detection involved successive conversion of acetylcholine into choline at the reaction electrode, then into betaine and hydrogen peroxide at the detection electrode. Reduction of hydrogen peroxide by hydrazine released electrons which were detected by chronoamperometry. The sensor exhibited a wide dynamic range (0.7 nM−1,500 μM), very low average LOD (0.6 nM), and high selectivity to acetylcholine from the multi-step reaction sequence. This sensor monitored the *in-vitro* extracellular release of acetylcholine by leukemic T-cells triggered by calcium ions. Aydin et al. (2018) utilized self-assembled films of a densely carboxylated PT derivative, poly(3-thiophene malonic acid) (P3-TMA, **9**), as electrochemical immunosensors for the protein Interleukin 1β (IL-1β) involved in human immune response. The polymer was synthesized by chemical oxidation of the methyl ester monomer, then hydrolysis yielding ionizable, carboxylic acid groups. These groups both bound to a treated ITO substrate with hydroxyl surface functionalities, forming a self-assembled P3-TMA monolayer, and immobilized anti-IL-1β antibodies on the monolayer permitting biorecognition. Selective immunodetection of IL-1β antigen in human serum and saliva was demonstrated with a very low LOD (3 fg/mL), two orders of magnitude lower than the next-best, CP-free system (300 fg/mL).

CP-based OECT configurations are gaining popularity as sensitive electrochemical biosensors. OECT biosensors commonly utilize water-processable poly(ethylenedioxythiophene):poly(styrene sulfonate) (PEDOT:PSS) (Liao et al., 2019), for example in conjunction with lipid bilayers to detect the ion pore α-hemolysin (Zhang et al., 2016). Recent OECT biosensors have explored alternative CPs with tailored structures. Giovannitti et al. (2018) synthesized a naphthalene-bithiophene-based donor-acceptor copolymer (10) and introduced glycol side chains *via* amine-anhydride coupling to improve ion transport in OECTs. The CP was coated with GOx or lactate oxidase without enzyme immobilization and used for glucose (Savva et al., 2019), or lactate (Pappa et al., 2018) detection, respectively, with good LODs (both 10 μM). Wustoni et al. (2019) synthesized thiophene derivatives functionalized with 15-crown-5 and 18-crown-6 ethers to selectively trap sodium and potassium ions, respectively, two important ions in cell signaling. Each monomer was copolymerized with ethylenedioxythiophene (EDOT) onto OECTs using various electropolymerization methods. Under an optimized OECT gate potential, the copolymers (11a/11b) performed selective, real-time detection of sodium and potassium ions with good LODs (20/100 μM), broad linear detection ranges (10–10^6^/100–10^6^ μM), and comparable sensitivities to the “gold-standard” PEDOT:PSS.

Alternatively to CPs, semiconducting dyes are commercially available and readily undergo electropolymerization forming redox-active polymers. The earlier use of organic dye-based polymers in electrochemical biosensors has been reviewed (Barsan et al., 2015). However, here we summarize the many recent biosensing applications of poly(methylene blue) (PMB, **12**), a prominent redox-active polymer, with one mode-1 and one mode-2 electrochemical biosensor. Dilgin et al. (2018) electropolymerized methylene blue onto poly(amidoamine)-coated, disposable graphite electrodes using CV, and immobilized glucose dehydrogenase (GDH) on the PMB with GA crosslinking. Aided by nicotinamide adenine dinucleotide (NAD^+^), this PMB-GDH system facilitated mode-2, amperometric glucose detection *via* several reactions. First, GDH oxidized glucose and reduced NAD^+^ to NADH; the underlying PMB then re-oxidized NADH; finally, an electrochemical potential re-oxidized PMB generating an electrical signal. This sensor selectively detected glucose in artificial blood serum and commercial dextrose solutions under flow conditions with a reasonable LOD (4.0 μM). Considering the disadvantages of enzymatic biosensors, including poor storage stability and high cost, Pandey et al. (2018) fabricated a non-enzymatic, mode-1 PMB biosensor for creatinine, an indicator of renal dysfunction. Dendritic PMB nanofibers were synthesized using CV onto a Cu-doped carbon nanofiber substrate, producing a polymer/metal/carbon nanocomposite sensor. Through PMB and creatine coordination to Cu centers, this sensor showed excellent selectivity, sensitivity (0.133 μA ng mL^−1^), and LOD (0.2 ng mL^−1^), with consistent measurements in clinical human saliva samples (1–2% RSD). Examples abound of recent electrochemical biosensors with polymers including PMB (Koyun and Sahin, 2018; Li et al., 2018; Wang and Ma, 2018; Bollella et al., 2019a,b), poly(alizarin yellow R) (Amini et al., 2019), poly(azure A) (Agrisuelas et al., 2018), poly(azure B) (Porfireva et al., 2019; Stoikov et al., 2019), poly(azure C) (Liu et al., 2019), poly(brilliant cresyl blue) (da Silva et al., 2019), and poly(thionine) (Shamspur et al., 2018; Wang Y. et al., 2018; Zhao and Ma, 2018; Chai and Kan, 2019; Stoikov et al., 2019), demonstrating the versatility of organic dyes in designing novel, semiconducting polymers for biosensors.

## Conclusions

Organic semiconducting polymers are a highly versatile, promising class of materials for biosensors since their organic synthesis can be tailored to achieve various functionalities for different applications. This versatility is evidenced by the numerous, innovative techniques in recent literature to synthesize organic biosensor materials. Electrochemical biosensors have especially great potential for widespread, *in-vivo* application, since recent biosensor materials exhibit high sensitivity, LODs as low as 0.0986 μM, and broad linear ranges spanning five orders of magnitude. Novel electrochemical transistor configurations including OECTs enhance sensitivity through signal amplification and represent a popular new direction in biosensor design. Mode-2 biosensors utilizing complex moieties are most common and historically well-established; here the organic semiconductor only transduces the electrochemical signal, while additional moieties including enzymes and bacteria are primarily responsible for detection. Consequently, numerous semiconductor functionalities have been developed to bind to these moieties, including amine groups for enzymes, and fused-ring backbones which also improve charge transfer. Developing chemical functionalities with improved enzyme binding may also reduce the current overdependence on GA crosslinking for enzyme adhesion.

Recent mode-1 biosensors, which directly mediate analyte redox, exhibit competitive LODs, and detection ranges compared to mode-2 biosensors (Table 1). Both mechanisms can achieve selectivity by introducing specific functionalities, including GOx for glucose, and boronic acids for dopamine. However, Pandey et al. (2018) note that mode-2 biosensors often suffer from reduced storage stability and higher cost from the additional, detecting moiety. Conversely, mode-1 biosensors are uncommon since they require the identification and synthesis of selective biosensing functionalities replacing naturally-occurring moieties. While both mechanisms are worth pursuing, future syntheses should also investigate novel semiconductors for mode-1 detection to complement established, “mode-2” technologies. Continued development of mode-1 biosensors would likely increase their impact in multiplying opportunities for low-cost, disposable devices for clinical use, especially when combined with “disposable” electrode materials including pencil graphite (Dilgin et al., 2018).

One material design strategy to expand the scope of mode-1 biosensors would involve introducing small molecule organic semiconductors such as spiropyran as pendant groups on aliphatic or conjugated polymers. As part of a growing trend toward small molecule semiconductors in biosensors, spiropyran derivatives are becoming increasingly common in optical biosensors due to their reversible photochromism under various stimuli. Recently, Li et al. (2013) utilized silyl-modified spiropyran for optical fluoride detection in biological media, while Shao et al. (2018) used siloxane polymers with spiropyran pendant groups for optical silver and iron(III) ion detection. Tao et al. (2016) extended the use of spiropyran derivatives to mode-1 electrochemical detection of fluoride. Future developments in synthesizing small molecule semiconductors as polymer pendant functionalities would help establish their viability in mode-1 electrochemical detection, expanding our ever-growing “library” of organic semiconductors for electrochemical biosensors.

## Author Contributions

All authors listed have made a substantial, direct and intellectual contribution to the work, and approved it for publication.

### Conflict of Interest

The authors declare that the research was conducted in the absence of any commercial or financial relationships that could be construed as a potential conflict of interest.

## References

[B1] Agrisuelas J. González-Sánchez M. I. Gómez-Monedero B. Valero E. (2018). A comparative study of poly(azure A) film-modified disposable electrodes for electrocatalytic oxidation of H_2_O_2_: effect of doping anion. Polymers 10:48 10.3390/polym10010048 PMC6414827 30966084

[B2] Akhtar M. H. Hussain K. K. Gurudatt N. G. Shim Y. B. (2017). Detection of Ca^2+^-induced acetylcholine released from leukemic T-cells using an amperometric microfluidic sensor. Biosens. Bioelectron. 98, 364–370. 10.1016/j.bios.2017.07.003 28704785

[B3] Alvarez A. Costa-Fernández J. M. Pereiro R. Sanz-Medel A. Salinas-Castillo A. (2011). Fluorescent conjugated polymers for chemical and biochemical sensing. Trends Anal. Chem. 30, 1513–1525. 10.1016/j.trac.2011.04.017

[B4] Amini N. Gholivand M. B. Shamsipur M. Movahedi A. A. M. Farahi S. Habibi-Rezaei M.. (2019). Fabrication of a glycation induced amyloid nanofibril and polyalizarin yellow R nanobiocomposite: application for electrocatalytic determination of hydrogen peroxide. Int. J. Biol. Macromol. 123, 1297–1304. 10.1016/j.ijbiomac.2018.10.043 30336241

[B5] Antonelli M. L. Spadaro C. Tornelli R. F. (2008). A microcalorimetric sensor for food and cosmetic analyses: l-Malic acid determination. Talanta 74, 1450–1454. 10.1016/j.talanta.2007.09.035 18371803

[B6] Aydin E. B. Aydin M. Sezgintürk M. K. (2018). Highly sensitive electrochemical immunosensor based on polythiophene polymer with densely populated carboxyl groups as immobilization matrix for detection of interleukin 1? in human serum and saliva. Sens. Actuat. B Chem. 270, 18–27. 10.1016/j.snb.2018.05.014

[B7] Azak H. Kurbanoglu S. Yildiz H. B. Ozkan S. A. (2016). Electrochemical glucose biosensing via new generation DTP type conducting polymers/gold nanoparticles/glucose oxidase modified electrodes. J. Electroanal. Chem. 770, 90–97. 10.1016/j.jelechem.2016.03.034

[B8] Azak H. Yildiz H. B. Bezgin Carbas B. (2018). Synthesis and characterization of a new poly(dithieno (3,2-b:2′, 3′-d) pyrrole) derivative conjugated polymer: its electrochromic and biosensing applications. Polymer 134, 44–52. 10.1016/j.polymer.2017.11.044

[B9] Bai L. Elósegui C. G. Li W. Yu P. Fei J. Mao L. (2019). Biological applications of organic electrochemical transistors: electrochemical biosensors and electrophysiology recording. Front. Chem. 7:313. 10.3389/fchem.2019.00313 31134185 PMC6514146

[B10] Barsan M. M. Ghica M. E. Brett C. M. A. (2015). Electrochemical sensors and biosensors based on redox polymer/carbon nanotube modified electrodes: a review. Anal. Chim. Acta 881, 1–23. 10.1016/j.aca.2015.02.059 26041516

[B11] Bhand S. G. Soundararajan S. Surugiu-Wärnmark I. Milea J. S. Dey E. S. Yakovleva M.. (2010). Fructose-selective calorimetric biosensor in flow injection analysis. Anal. Chim. Acta 668, 13–18. 10.1016/j.aca.2010.01.020 20457296

[B12] Bollella P. Sharma S. Cass A. E. G. Antiochia R. (2019a). Microneedle-based biosensor for minimally-invasive lactate detection. Biosens. Bioelectron. 123, 152–159. 10.1016/j.bios.2018.08.010 30177422

[B13] Bollella P. Sharma S. Cass A. E. G. Antiochia R. (2019b). Minimally-invasive microneedle-based biosensor array for simultaneous lactate and glucose monitoring in artificial interstitial fluid. Electroanalysis 31, 374–382. 10.1002/elan.201800630

[B14] Buber E. Soylemez S. Udum Y. A. Toppare L. (2018). Fabrication of a promising immobilization platform based on electrochemical synthesis of a conjugated polymer. Colloids Surf. B. Biointerfaces 167, 392–396. 10.1016/J.COLSURFB.2018.04.041 29702470

[B15] Casado N. Hernández G. Sardon H. Mecerreyes D. (2016). Current trends in redox polymers for energy and medicine. Prog. Polym. Sci. 52, 107–135. 10.1016/j.progpolymsci.2015.08.003

[B16] Cervera J. Pietak A. Levin M. Mafe S. (2018). Bioelectrical coupling in multicellular domains regulated by gap junctions: a conceptual approach. Bioelectrochemistry 123, 45–61. 10.1016/j.bioelechem.2018.04.013 29723806

[B17] Cevik E. Cerit A. Tombuloglu H. Sabit H. Yildiz H. B. (2019). Electrochemical glucose biosensors: whole cell microbial and enzymatic determination based on 10-(4H-Dithieno[3,2-b:2′,3′-d]Pyrrol-4-yl)Decan-1-amine interfaced glassy carbon electrodes. Anal. Lett. 52, 1138–1152. 10.1080/00032719.2018.1521828

[B18] Chai R. Kan X. (2019). Au-polythionine nanocomposites: a novel mediator for bisphenol A dual-signal assay based on imprinted electrochemical sensor. Anal. Bioanal. Chem. 411, 3839–3847. 10.1007/s00216-019-01858-3 31123779

[B19] Chen J. Burrell A. K. Campbell W. M. Officer D. L. Too C. O. Wallace G. G. (2004). Photoelectrochemical cells based on a novel porphyrin containing light harvesting conducting copolymer. Electrochim. Acta 49, 329–337. 10.1016/j.electacta.2003.08.015

[B20] Clark L. C. Jr. Lyons C. (1962). Electrode systems for continuous monitoring in cardiovascular surgery. Ann. N. Y. Acad. Sci. 102, 29–45. 10.1111/j.1749-6632.1962.tb13623.x 14021529

[B21] da Silva W. Ghica M. E. Brett C. M. A. (2019). Novel nanocomposite film modified electrode based on poly(brilliant cresyl blue)-deep eutectic solvent/carbon nanotubes and its biosensing applications. Electrochim. Acta 317, 766–777. 10.1016/j.electacta.2019.06.003

[B22] Dervisevic M. Senel M. Cevik E. (2017a). Novel impedimetric dopamine biosensor based on boronic acid functional polythiophene modified electrodes. Mater. Sci. Eng. C 72, 641–649. 10.1016/j.msec.2016.11.127 28024633

[B23] Dervisevic M. Senel M. Sagir T. Isik S. (2017b). Highly sensitive detection of cancer cells with an electrochemical cytosensor based on boronic acid functional polythiophene. Biosens. Bioelectron. 90, 6–12. 10.1016/j.bios.2016.10.100 27866080

[B24] Dilgin D. G. Ertek B. Dilgin Y. (2018). A low-cost, fast, disposable and sensitive biosensor study: flow injection analysis of glucose at poly-methylene blue-modified pencil graphite electrode. J. Iran. Chem. Soc. 15, 1355–1363. 10.1007/s13738-018-1335-x

[B25] Esmer E. N. Tarkuc S. Udum Y. A. Toppare L. (2011). Near infrared electrochromic polymers based on phenazine moieties. Mater. Chem. Phys. 131, 519–524. 10.1016/j.matchemphys.2011.10.014

[B26] Fidanovski K. Mawad D. (2019). Conjugated polymers in bioelectronics: addressing the interface challenge. Adv. Healthc. Mater. 8, 1–9. 10.1002/adhm.201900053 30941922

[B27] Gaddes D. Reeves W. B. Tadigadapa S. (2017). Calorimetric biosensing system for quantification of urinary creatinine. ACS Sens. 2, 796–802. 10.1021/acssensors.7b00161 28723128

[B28] Giovannitti A. Maria I. P. Hanifi D. Donahue M. J. Bryant D. Barth K. J.. (2018). The role of the side chain on the performance of N-type conjugated polymers in aqueous electrolytes. Chem. Mater. 30, 2945–2953. 10.1021/acs.chemmater.8b00321 29780208 PMC5953566

[B29] Hopkins J. Travaglini L. Lauto A. Cramer T. Fraboni B. Seidel J. (2019). Photoactive organic substrates for cell stimulation: progress and perspectives. Adv. Mater. Technol. 4:1800744 10.1002/admt.201800744

[B30] Jiang K. Wang Y. Thakur G. Kotsuchibashi Y. Naicker S. Narain R.. (2017). Rapid and highly sensitive detection of dopamine using conjugated oxaborole-based polymer and glycopolymer systems. ACS Appl. Mater. Interfaces 9, 15225–15231. 10.1021/acsami.7b04178 28437064

[B31] Katrlík J. Voštiar I. Šefčovičová J. Tkáč J. Mastihuba V. Valach M.. (2007). A novel microbial biosensor based on cells of Gluconobacter oxydans for the selective determination of 1,3-propanediol in the presence of glycerol and its application to bioprocess monitoring. Anal. Bioanal. Chem. 388, 287–295. 10.1007/s00216-007-1211-5 17393157

[B32] Kim D. M. Yoon J. H. Won M. S. Shim Y. B. (2012). Electrochemical characterization of newly synthesized polyterthiophene benzoate and its applications to an electrochromic device and a photovoltaic cell. Electrochim. Acta 67, 201–207. 10.1016/j.electacta.2012.02.033

[B33] Koyun O. Sahin Y. (2018). Voltammetric determination of nitrite with gold nanoparticles/poly(methylene blue)-modified pencil graphite electrode: application in food and water samples. Ionics 24, 3187–3197. 10.1007/s11581-017-2429-7

[B34] Li G. Feng X. Fei J. Cai P. Li J. Huang J. (2017). Interfacial assembly of photosystem II with conducting polymer films toward enhanced photo-bioelectrochemical cells. Adv. Mater. Interfaces 4:1600919 10.1002/admi.201600619

[B35] Li Y. Duan Y. Zheng J. Li J. Zhao W. Yang S.. (2013). Self-assembly of graphene oxide with a silyl-appended spiropyran dye for rapid and sensitive colorimetric detection of fluoride ions. Anal. Chem. 85, 11456–11463. 10.1021/ac402592c 24164279

[B36] Li Y. He J. Chen J. Niu Y. Zhao Y. Zhang Y.. (2018). A dual-type responsive electrochemical immunosensor for quantitative detection of PCSK9 based on n-C60-PdPt/N-GNRs and Pt-poly (methylene blue) nanocomposites. Biosens. Bioelectron. 101, 7–13. 10.1016/j.bios.2017.09.043 29031129

[B37] Liao J. Si H. Zhang X. Lin S. (2019). Functional sensing interfaces of PEDOT:PSS organic electrochemical transistors for chemical and biological sensors: a mini review. Sensors 19:218. 10.3390/s19020218 30634408 PMC6359468

[B38] Liu Y. Song N. Ma Z. Zhou K. Gan Z. Gao Y. (2019). Synthesis of a poly(N-methylthionine)/reduced graphene oxide nanocomposite for the detection of hydroquinone. Mater. Chem. Phys. 223, 548–556. 10.1016/j.matchemphys.2018.11.045

[B39] Miyagishi H. V. Tamaki T. Masai H. Terao J. (2019). Synthesis and acid-responsiveness of an insulated π-conjugated polymer containing spiropyrans in its backbone. Molecules 24:1301. 10.3390/molecules24071301 30987095 PMC6480293

[B40] Moon J. M. Thapliyal N. Hussain K. K. Goyal R. N. Shim Y. B. (2018). Conducting polymer-based electrochemical biosensors for neurotransmitters: a review. Biosens. Bioelectron. 102, 540–552. 10.1016/j.bios.2017.11.069 29220802

[B41] Naveen M. H. Gurudatt N. G. Shim Y. B. (2017). Applications of conducting polymer composites to electrochemical sensors: a review. Appl. Mater. Today 9, 419–433. 10.1016/j.apmt.2017.09.001

[B42] Oliveira-Brett A. M. Diculescu V. C. Enache T. A. Fernandes I. P. G. Chiorcea-Paquim A. M. Oliveira S. C. B. (2019). Bioelectrochemistry for sensing amino acids, peptides, proteins and DNA interactions. Curr. Opin. Electrochem. 14, 173–179. 10.1016/j.coelec.2019.03.008

[B43] Pandey I. Bairagi P. K. Verma N. (2018). Electrochemically grown polymethylene blue nanofilm on copper-carbon nanofiber nanocomposite: an electrochemical sensor for creatinine. Sens. Actuat. B. Chem. 277, 562–570. 10.1016/j.snb.2018.09.036

[B44] Pappa A. M. Ohayon D. Giovannitti A. Maria I. P. Savva A. Uguz I.. (2018). Direct metabolite detection with an n-type accumulation mode organic electrochemical transistor. Sci. Adv. 4, 1–8. 10.1126/sciadv.aat0911 29942860 PMC6014717

[B45] Parameswaran M. Balaji G. Jin T. M. Vijila C. Vadukumpully S. Furong Z. (2009). Charge transport studies in fluorene - Dithieno[3,2-b:2′,3′-d]pyrrole oligomer using time-of-flight photoconductivity method. Org. Electron. Phys. Mater. Appl. 10, 1534–1540. 10.1016/j.orgel.2009.08.022

[B46] Park D.-S. Shim Y.-B. Rahman M. A. Kumar P. (2008). Electrochemical sensors based on organic conjugated polymers. Sensors 8, 118–141. 10.3390/s8010118 27879698 PMC3681146

[B47] Pohanka M. (2017). The piezoelectric biosensors: principles and applications, a review. Int. J. Electrochem. Sci. 12, 496–506. 10.20964/2017.01.44

[B48] Porfireva A. Vorobev V. Babkina S. Evtugyn G. (2019). Electrochemical sensor based on Poly(Azure B)-DNA composite for doxorubicin determination. Sensors 19, 1–14. 10.3390/s19092085 31060322 PMC6539792

[B49] Rasmussen S. C. Evenson S. J. (2013). Dithieno[3,2-b:2′,3′-d]pyrrole-based materials: synthesis and application to organic electronics. Prog. Polym. Sci. 38, 1773–1804. 10.1016/j.progpolymsci.2013.04.004

[B50] Savva A. Ohayon D. Surgailis J. Paterson A. F. Hidalgo T. C. Chen X. (2019). Solvent engineering for high-performance n-type organic electrochemical transistors. Adv. Electron. Mater. 5:1900249 10.1002/aelm.201900249

[B51] Shamspur T. Biniaz Z. Mostafavi A. Torkzadeh-Mahani M. Mohamadi M. (2018). An electrochemical immunosensor based on poly(thionine)-modified carbon paste electrode for the determination of prostate specific antigen. IEEE Sens. J. 18, 4861–4868. 10.1109/JSEN.2018.2832083

[B52] Shao Z. Dai X. Li H. Zhao Y. Xu Z. Zheng T. (2018). Multiresponsive polysiloxane bearing spiropyran: synthesis and sensing of pH and metal ions of different valence. Mater. Res. Express 5:35301 10.1088/2053-1591/aab11e

[B53] Skládal P. (2016). Piezoelectric biosensors. Trends Anal. Chem. 79, 127–133. 10.1016/j.trac.2015.12.009

[B54] Soylemez S. Kaya H. Z. Udum Y. A. Toppare L. (2019). A multipurpose conjugated polymer: electrochromic device and biosensor construction for glucose detection. Org. Electron. 65, 327–333. 10.1016/J.ORGEL.2018.11.001

[B55] Stoikov D. I. Porfir'eva A. V. Shurpik D. N. Stoikov I. I. Evtyugin G. A. (2019). Electrochemical DNA sensors on the basis of electropolymerized thionine and Azure B with addition of pillar[5]arene as an electron transfer mediator. Russ. Chem. Bull. 68, 431–437. 10.1007/s11172-019-2404-8

[B56] Tao J. Zhao P. Li Y. Zhao W. Xiao Y. Yang R. (2016). Fabrication of an electrochemical sensor based on spiropyran for sensitive and selective detection of fluoride ion. Anal. Chim. Acta 918, 97–102. 10.1016/j.aca.2016.03.025 27046215

[B57] Udum Y. A. Yildiz H. B. Azak H. Sahin E. Talaz O. Çirpan A. (2014). Synthesis and spectroelectrochemistry of dithieno(3,2-b:2′,3′- d)pyrrole derivatives. J. Appl. Polym. Sci. 131, 8676–8683. 10.1002/app.40701

[B58] Wallace G. G. Too C. O. Officer D. L. Dastoor P. C. (2005). Photoelectrochemical cells based on inherently conducting polymers. MRS Bull. 30, 46–49. 10.1557/mrs2005.9

[B59] Wang H. Ma Z. (2018). A novel strategy for improving amperometric biosensor sensitivity using dual-signal synergistic effect for ultrasensitive detection of matrix metalloproteinase-2. Sens. Actuat. B Chem. 266, 46–51. 10.1016/j.snb.2018.03.119

[B60] Wang J. Lv F. Liu L. Ma Y. Wang S. (2018). Strategies to design conjugated polymer based materials for biological sensing and imaging. Coord. Chem. Rev. 354, 135–154. 10.1016/j.ccr.2017.06.023

[B61] Wang N. Yang A. Fu Y. Li Y. Yan F. (2019). Functionalized organic thin film transistors for biosensing. Acc. Chem. Res. 52, 277–287. 10.1021/acs.accounts.8b00448 30620566

[B62] Wang Y. Jiang M. Shan Y. Jin X. Gong M. Wang X. (2018). Nano polythionine-based electrochemiluminescence biosensor for detection of the p16INK4a gene using RuAg@AuNPs core-shell nanocomposites as DNA labels. J. Lumin. 201, 135–142. 10.1016/j.jlumin.2018.04.039

[B63] White H. S. Kittlesen G. P. Wrighton M. S. (1984). Chemical derivatization of an array of three gold microelectrodes with polypyrrole: fabrication of a molecule-based transistor. J. Am. Chem. Soc. 106, 5375–5377. 10.1021/ja00330a070

[B64] Wu F. Yu P. Mao L. (2017). Bioelectrochemistry for in vivo analysis: interface engineering toward implantable electrochemical biosensors. Curr. Opin. Electrochem. 5, 152–157. 10.1016/j.coelec.2017.08.008

[B65] Wustoni S. Combe C. Ohayon D. Akhtar M. H. McCulloch I. Inal S. (2019). Membrane-free detection of metal cations with an organic electrochemical transistor. Adv. Funct. Mater. [Epub ahead of print]. 10.1002/adfm.201904403

[B66] Zhang Y. Inal S. Hsia C. Y. Ferro M. Ferro M. Daniel S. (2016). Supported lipid bilayer assembly on PEDOT:PSS films and transistors. Adv. Funct. Mater. 26, 7304–7313. 10.1002/adfm.201602123

[B67] Zhao L. Ma Z. (2018). Facile synthesis of polyaniline-polythionine redox hydrogel: conductive, antifouling and enzyme-linked material for ultrasensitive label-free amperometric immunosensor toward carcinoma antigen-125. Anal. Chim. Acta 997, 60–66. 10.1016/j.aca.2017.10.017 29149995

